# Cultural Approaches to Addressing Sleep Deprivation and Improving Sleep Health in Japan: Sleep Issues Among Children and Adolescents Rooted in Self-Sacrifice and Asceticism

**DOI:** 10.3390/children12050566

**Published:** 2025-04-27

**Authors:** Jun Kohyama

**Affiliations:** Department of Pediatrics, Tokyo Bay Urayasu Ichikawa Medical Center, Urayasu 279-0001, Japan; info@j-kohyama.jp; Tel.: +81-47-351-3101

**Keywords:** Bushidō, optimal sleep duration, sleep health literacy, Yōjōkun, seppuku, kamikaze pilots

## Abstract

This narrative review examines the issue of sleep deprivation among children and adolescents in Japan, exploring its cultural origins and evaluating the current state of sleep education and interventions. It emphasizes the profound influence of the Bushidō spirit, with its focus on self-sacrifice and asceticism, as a core factor in the undervaluation of sleep in Japanese society. While educational initiatives and interventions highlighting the importance of sleep exist, significant limitations remain in improving sleep habits. Sleep deprivation continues to affect children and adolescents, despite its considerable impact on mental health and academic performance. This review presents a method for personalized sleep duration estimation and assesses its potential impact on improving sleep health by using optimal sleep duration calculations. The review also proposes practical steps to improve sleep duration through individualized strategies, integrating cultural context to mitigate the serious health risks associated with insufficient sleep. Ultimately, it underscores the need for targeted strategies to improve sleep among children and adolescents—particularly through personalized optimal sleep duration estimation—while advocating for a shift in cultural perspective beyond self-sacrifice and asceticism. The review highlights the importance of cultural transformation and suggests future research directions and practical applications.

## 1. Introduction

“To all those who sacrifice sleep to keep fighting”. This phrase appeared in a pillow advertisement in 2024 [1]. A similar message, found on beverages sold to elementary and junior high school students since 2000 and reconsidered in 2016, read, “Keep going, even when you’re tired! For elementary and junior high students” [2]. Additionally, the famous slogan for a nutritional drink released in 1988, “Can you fight for 24 h?” became widely popular in 1989 [3]. From the perspective of sleep health literacy [4], these slogans that have emerged in Japan are clearly problematic. In fact, Japan is one of the leading countries for short sleep duration [5], including among children [6]. Despite numerous reports highlighting the challenges posed by sleep deprivation [7,8], why do such unusual slogans continue to circulate in Japan, and why has the issue of short sleep duration remained unresolved? In Japan, the number of sleep-related products and discussions about sleep has rapidly increased, with new articles about sleep appearing online daily. However, the style in which these issues are reported often lacks depth, giving the impression that sleep is being undervalued. Is this concern an overreaction, or does it reflect a more profound, systemic issue?

This review begins with an overview of current sleep education practices and then delves into the historical and cultural roots of sleep undervaluation in Japan. The author undertakes this examination out of concern that, without understanding the underlying cultural causes that perpetuate short sleep, the situation will remain unchanged. Subsequently, this review introduces a simple method for determining an individual’s optimal sleep duration (OSD), recently reported for children and adolescents, as a concrete solution to their sleep problems.

## 2. Sleep Education

The importance of sleep must be considered in terms of both quantity and quality [9]. In particular, the impact of sleep duration on mental health and quality of life is more pronounced in adolescents than in older adults [10], highlighting the critical need to secure OSD during this developmental stage. Furthermore, sleep quality in early adolescence has been shown to (1) significantly correlate with mental distress and (2) influence the development of white matter pathways, such as the uncinate fasciculus, which connects the amygdala and prefrontal cortex. These findings suggest that poor sleep quality in early adolescence may play a pivotal role in the onset of anxiety disorders [11]. Therefore, special attention must be directed toward the sleep of children and adolescents, with a comprehensive focus on both sleep quantity and quality, as emphasized by numerous experts [12,13,14,15]. Sleep problems among children and adolescents have been linked to various unhealthy lifestyle behaviors, including skipping breakfast, frequent consumption of fast food and sugary snacks, and increased screen time [16,17,18]. According to Perez Zarate et al. [19], insufficient sleep among high school students is primarily attributable to academic and extracurricular burdens, as well as the use of electronic devices at night. In the author’s own study involving 2722 students from 5th to 12th grade, several factors were found to be significantly associated with decreased average sleep duration, including longer weekday screen time, irregular dinner habits, extended after-school activity duration, poor defecation habits, and higher physical activity [20]. Moreover, shorter sleep duration before school days was independently associated with skipping breakfast [21]. Luo et al. reported that adequate daily outdoor activity time is linked to a decreased risk of insufficient sleep among children aged 6 to 17 years [22]. While Macharla et al. [23] acknowledged the potential benefits of smartphones in terms of social connectivity, educational applications, and financial tools, they also emphasized the critical importance of counseling adolescents on the adverse effects of smartphone use before bedtime. Taken together, numerous studies underscore the urgent need to implement effective intervention programs to combat sleep deprivation among youth [19,23,24,25]. Indeed, many school-based sleep education programs published since 2020 (all from outside Japan) have successfully improved students’ knowledge about sleep [26,27,28,29,30,31,32,33,34,35]. However, with few exceptions [32,33,34,35], these programs have largely failed to produce meaningful behavioral changes regarding sleep, likely due to the academic and extracurricular pressures placed on students [19]. A systematic review [36] also concluded that school-based solutions failed to improve sleep health. Given the limited effectiveness of simply disseminating knowledge in addressing sleep deprivation [26,27,28,29,30,31,36], it may be essential to explore strategies aimed at reducing the burdens imposed on students. These burdens are often shaped by cultural factors. For instance, the intense competition surrounding university entrance exams is well documented in countries like China [37] and South Korea [38]. Such pressures are deeply embedded in societal values and the attitudes of educators, which significantly influence students’ behaviors. Furthermore, as discussed in the following “Section 4 strategies”, there is an expectation that the internalization of positive experiences will play a key role in this approach.

In Japan, the number of children and adolescents with disrupted circadian rhythms has increased significantly [4,39,40]. While some studies have reported modest positive effects of sleep education on children [41] and adolescents [42], the overall impact remains limited. Although Japan’s 2018 work style reform legislation introduced a “working interval regulation” [43], by 2024, over 80% of companies still had no plans to implement it [44]. A television program broadcast on 11 June 2024—Suffering Bureaucrats: What is Happening in the Heart of the Japanese Government?—highlighted the excessive overtime faced by government officials [45].

This issue extends beyond the corporate and governmental sectors to include the education system. Teachers, frequently subjected to long working hours due to a culture of self-sacrifice, serve as role models for children and adolescents, who may consequently adopt similar lifestyle patterns [46]. The author expresses concern that this deeply rooted cultural norm of overwork, grounded in self-sacrifice, may be passed down intergenerationally. Without structural intervention, sleep will continue to be undervalued in Japanese society, and this trend is likely to persist. Alarmingly, limited sleep literacy is not confined to Japan. For instance, even occupational therapists in other countries have demonstrated insufficient knowledge regarding sleep assessment and practical interventions [47].

## 3. Perception of Sleep in Japan

### 3.1. Sleep in Japan Before the Emergence of Yōjōkun

In classical Japanese literature, terms such as “nemachizuki” and “fushimachizuki” were used to indicate bedtime. These elegant expressions refer to the moon visible four days after the full moon in the eighth month of the lunar calendar (the 19th day of the moon) [48]. The names are believed to have originated from the custom of going to bed when the moon rose in the evening. The earliest record of “nemachizuki” appears in the 25th chapter of Tōtō Nikki, dating back to 957 AD [49]. According to calculations by the National Astronomical Observatory of Japan [50], the moonrise on the 19th day of the eighth lunar month in 957 occurred at 20:32, suggesting that bedtime during the Heian period for the aristocracy was approximately 2.6 h after sunset, which occurred at 17:56.

In the book Early Rising [51], the warrior Tōdō Takatora (1556–1630), who was active during the Sengoku and early Edo periods, wrote, “One should rest by 8 p.m.”. Furthermore, a passage from the book Bushikokoroe advises, “One should go to bed early at night”. This book is thought to have been written in the early Edo period, when the health benefits of sleep were widely emphasized, similar to descriptions in the Korean medical text Dongui Bogam, which was well known in Japan [52]. During this period, there seems to have been no widespread tendency to undervalue sleep.

### 3.2. Yōjōkun

Yōjōkun is a widely known text in Japan [53]. The author, Kaibara Ekiken (1630–1714), was a Confucian scholar based in Fukuoka during the Edo period. Written in 1712 at the age of 83, Yōjōkun draws from Ekiken’s own experiences. The philosophy of Yōjōkun explores methods for promoting physical and mental health and achieving longevity. Its intellectual roots are traced to the natural philosophy of Laozi, and its fundamental principles echo the opening lines of the Huangdi Neijing Suwen, which state, “Eating and drinking in moderation, maintaining regularity in daily activities, and avoiding excessive labor” [54].

In Chapter 23 of Huangdi Neijing Suwen, “Xuanming Wuqi Pian”, it is written that “Excessive sleep depletes the qi”, warning against oversleeping. This caution aligns with recent epidemiological studies, which have revealed that insufficient sleep increases the risks of obesity, ischemic heart disease, cerebrovascular disorders, type 2 diabetes, hypertension, and mortality [55,56]. On the other hand, excessive sleep has also been associated with higher risks of these conditions and mortality [56,57]. While the exact reasons for this relationship remain unclear, achieving an individual’s OSD minimizes the risks of illness and death. In Section 2, “Si Qi Tiao Shen Da Lun”, sleep patterns according to the seasons are recommended. In spring and summer, it suggests a later bedtime and earlier wake-up time, while in autumn, one should go to bed early and wake up early. In winter, bedtime should be slightly earlier, and wake-up time later, aligning with sunrise and sunset. This advice aligns with a study conducted on 55,000 individuals in Germany, which showed that sleep duration fluctuates with the seasons, being 20 min longer in winter than in summer [58]. Additionally, research on hunting–gathering societies in Africa and South America found that they woke up before dawn and went to bed approximately 3.3 h after sunset [59]. Consequently, sleep duration in winter was about one hour longer than in summer. Gokhale et al. [60] reviewed the association between the moon and health, noting a significantly longer sleep duration during the full moon phase compared to the new moon phase, supported by both subjective [61] and objective [62] measures. However, other studies [63] indicated no significant impact of the lunar cycle on human sleep. Although the Huangdi Neijing does not mention any connection between lunar phases and sleep, it fundamentally reflects natural human tendencies, emphasizing the importance of adequate sleep.

In contrast, Yōjōkun is often interpreted as contributing to the undervaluation of sleep. To put it drastically, the underlying message of Yōjōkun is “Do not sleep”. Kaibara Ekiken, who served the Kuroda clan, also lectured on Confucianism. During the early Edo period, the concept of explaining the lord–servant relationship through Zhu Xi’s Confucianism was established by figures like Yamaga Sokō. Confucianism emphasizes asceticism and spiritual cultivation, and it is believed that this asceticism is reflected in Yōjōkun. Yōjōkun, while deeply embedded in Confucian philosophy, served as a vehicle for promoting self-discipline and work ethic, often at the expense of health-related practices such as sufficient sleep.

In Yōjōkun, sleep is mentioned 16 times across eight volumes. It is noted that overeating, excessive drinking, and excessive sleep can harm one’s health. Volume 1, Section 24 of Yōjōkun, warns, “Excessive sleep causes illness and shortens life”, and Section 28 of Yōjōkun encourages reducing sleep to improve health, asserting that the need for sleep decreases with willpower. Kaibara Ekiken seems to view sleep as a passive activity, not acknowledging its active role. In contrast, in Huangdi Neijing’s Ling Shu, sleep is recognized not merely as a countermeasure against wakefulness but also as an active process that requires energy (qi). Volume 2, Section 1 of Huangdi Neijing’s Ling Shu, states, “Do not lie down and relax after meals. Resting or sleeping for long periods will disturb your mood and lead to illness”.

Why did Yōjōkun adopt such a dismissive stance toward sleep? One important factor is that Ekiken belonged to the samurai class. The samurai, a group whose profession was warfare, developed the concept of “Bushidō” (the Way of the Warrior) [64]. The term Bushidō is first recorded in the early Edo period in Kōyō Gunkan [65]. With the advent of the Edo period, intense warfare subsided, and the samurai no longer needed to refine their combat skills. As a result, the samurai spirit shifted from martial techniques to Bushidō, which became increasingly refined. Bushidō is characterized by the spirit of self-sacrifice, exemplified by the practice of seppuku (ritual suicide). The ideology of the ruling class gradually permeated the common people. Yōjōkun, which incorporates not only the principles of Zhu Xi’s Confucianism but also the spirit of Bushidō, likely spread to the general population.

### 3.3. From Yōjōkun to the End of World War II

While early Edo-period writings such as Yōjōkun prioritized moral discipline over physical health, the Meiji period, including the late Edo period, marked a shift towards modernized views on health, though traces of the earlier ascetic values persisted.

The 1832 publication Byōke Suchi [66], Japan’s first home medical encyclopedia and guide for home nursing, recommends going to bed early at night and rising before sunrise. This advice reflects a more balanced view of sleep, contrasting with the spiritual perspective of Yōjōkun and suggesting a shift in views on sleep in broader society.

In Hito to Tegami [67], written about the author of Nanso Satomi Hakkenden, Takizawa Bakin (1767–1848), it is stated that “Bakin and his family strictly adhered to a bedtime of 10 p.m. for many years”. Additionally, Bakin’s diary records, “Due to today’s heat, I stopped writing and spent the day reading. I went to bed around 8 p.m.”. This suggests that Bakin’s habits were not aligned with the principles in Yōjōkun.

The Iyo History Association website [68] introduces a diary written by Saionji Genjūrō, a figure involved in the Uwajima Domain during the late Edo period. According to this diary, during six days in June, the individual did not stay awake past 10 p.m., indicating that late Edo society was not universally affected by sleep deprivation, contrary to the warnings of Yōjōkun about excessive sleep.

During the Meiji period, prominent figures such as Fukuzawa Yukichi were known to go to bed by 10 p.m. In 1912, a popular elementary school song, Mura no Kajiya, depicted a blacksmith maintaining his health through regular sleep patterns [69]. Furthermore, the rules of the Early Rising Youth Association, founded in 1920, encouraged waking up early while emphasizing the importance of securing 8 h of sleep for health maintenance [51].

In the late Meiji period, with the advancement of modernization, Ninomiya Sontoku (also known as Kanjiro) became an influential figure regarding sleep. He is depicted in elementary school choral songs [70] and morality textbooks [71], embodying perseverance, diligence, and self-sacrifice through late-night studying and early rising. However, these portrayals lacked biological or empirical evidence regarding circadian rhythms or sunlight’s effects on sleep cycles. Instead, they reflected Confucian and Bushidō-based ascetic ideologies, further perpetuating the undervaluation of sleep by associating laziness with poor moral character.

### 3.4. After World War II

Before World War II, asceticism based on Confucian principles and Bushidō was strongly emphasized in Japan. Following the country’s defeat in the war, a backlash was expected, but the 1948 school anthem “Song of Youth” included the phrase “Funrei kokku”, meaning “strive with effort” [72]. This phrase reflects the power of will emphasized by Mencius. In Confucianism, personal moral cultivation and academic achievement were prioritized, and Japan, influenced by these values, continued to reduce sleep hours even after the war. As symbolized by the 1989 slogan “Can you fight for 24 h?” [3], Japan rapidly advanced toward becoming an economic powerhouse, with the spirit of Bushidō playing a key role.

On 27 August 2009, the headline of a news article read, “Personal Reasons: Japan Glass, President Resigns”. Mr. Chambers said in a press conference, “I have decided to put family first and company second”. He acknowledged that this decision might go against the prevailing social norms in Japan, where it is common for workers to prioritize the company above all else. “In that process, I have learned that I am not Japanese”, he remarked [73]. This statement offers a glimpse into the societal culture in Japan, which often imposes self-sacrifice on individuals.

The tragic case of Matsuri Takahashi, a Dentsu employee who died by suicide due to overwork in 2015 [74], has become an important theme in my lectures at the women’s university. Reflecting on Matsuri’s case, especially when observing students of a similar age, I am always strongly reminded that we must not let history repeat itself. One student’s reflection after a lecture noted the pressures that new employees face, the sense of responsibility toward their work, and the inability to ask for help. The student also mentioned that living alone, like Matsuri, can lead to being overlooked and not receiving support, which could result in similar tragic outcomes. This response highlights the importance of mental health support systems in the workplace. Below is an excerpt from a student’s writing: “The headline of a reference article, ‘You may be pushing someone to their limits’ [75], was shocking. I was surprised to learn that 90,000 people a year are involved in re-delivery, with approximately 10% of delivery workers spending time on it. Also, the words of a former Dentsu employee resonated with me: ‘Clients are treated like “gods”, while staff and subcontractors meet unreasonable demands through inhumane efforts. But they are also human. Even seemingly trivial demands can take someone’s time and push them to their limits. Everyone should imagine that”.

A survey conducted in 2017 found that the most desired activity among high school students involved in sports clubs is “to sleep” [76]. This result, when interpreted in reverse, reflects a culture where sports club activities and, likely, academic work are prioritized over sleep, embodying the cultural norm of “sacrificing sleep to engage in other activities”.

### 3.5. Summary of Section 3

Looking at the history of sleep undervaluation in Japan, it becomes clear that this attitude is deeply rooted in the aesthetic of self-sacrifice and asceticism, first exemplified in Yōjōkun. It is posited that Yōjōkun represents an early manifestation of Japan’s enduring tendency to undervalue sleep in contemporary society. While there have been periods of acknowledgment of sleep’s importance, the prevailing attitude in Japanese society has often been that sleep is a form of laziness. This cultural perspective, not solely shaped by Yōjōkun, emerges from Japan’s unique metaphysical aesthetics—specifically the cultural acceptance of self-sacrifice and asceticism. A similar argument is put forward by Miura [77], who states, “The emperor’s decision to end the war without being held accountable allowed Japan to place responsibility on the government and military leaders, fostering a collective view of Japan as a war victim rather than an aggressor. The kamikaze pilots came to symbolize Japan’s self-sacrifice during the war, a principle rooted in Bushidō. After the war, this ethos was criticized for diminishing the value of individual life. Nevertheless, the spirit of self-sacrifice persisted, transitioning from a military context to economic reconstruction. While Japan no longer demands the sacrifice of life for the state, the ethos of self-sacrifice for family and company remains deeply ingrained”. Transforming these deeply rooted cultural values is challenging, but recognizing this cultural background is essential to addressing the harmful effects of sleep deprivation.

## 4. Strategies

This section will propose strategies to improve insufficient sleep among children and adolescents, incorporating not only Japanese cultural elements but also global perspectives. This review emphasizes that the Bushidō spirit, which values self-sacrifice and asceticism, constitutes a significant barrier to effective strategies for mitigating sleep deprivation in Japan. The cultural glorification of self-sacrifice, often associated with maintaining productivity at the expense of personal well-being, fosters a societal undervaluation of sleep. To address this issue, it is crucial to analyze the underlying cultural causes. Understanding these cultural factors is essential for developing effective, culturally sensitive solutions that can resonate within the context of Japanese society. This approach does not call for the rejection of the ethical principles inherent in Bushidō, but rather underscores the importance of critically examining their impact on sleep health. A shift in the mindset of educators and caregivers is urgently needed, especially regarding the harmful cultural implications of excessively glorifying Bushidō. The following proposes five critical steps (Table 1) aimed at fostering a deeper awareness that transcends the ideals of self-sacrifice and asceticism to reduce sleep deprivation in Japan. These steps may also be applicable to children and adolescents in other cultural contexts.

1. Step 1: Increase knowledge about the broad range of negative impacts caused by sleep deprivation. In Japan, where societal pressures to sacrifice sleep for work and academic performance are deeply embedded in cultural practices, raising awareness of the detrimental effects of sleep deprivation can be a powerful first step. Acknowledging that chronic sleep deprivation negatively affects cognitive function, mental health, academic performance, and physical health is particularly important in a culture that often prioritizes work over rest. Recent studies highlight the specific adverse impacts, including impaired cognitive function [78], poor academic performance [79], increased behavioral problems [80], mental instability [81], risk of accidents [82], obesity [83], and negative effects on the cardiovascular [84], immune [85], and metabolic systems [86].

Maric et al. [87] examined the effects of chronic sleep deprivation (5 h of sleep for 7 days) and acute sleep deprivation (40 h of continuous wakefulness) on risk-taking behavior and brainwave activity among young men. The findings suggested that chronic sleep deprivation increases risk-taking behavior and leads to a decrease in delayed wave amplitude in the right prefrontal cortex, indicating that chronic sleep deprivation adversely affects decision-making. Furthermore, sleep deprivation can lead to distractibility, diminished executive function, difficulty in emotional regulation (including increased suicidal ideation), increased engagement in health-risk behaviors such as alcohol and drug use, and a higher risk of accidents and sports-related injuries [8]. In 2024, the author introduced seven key points for achieving sleep health literacy [88].

However, knowledge alone does not necessarily translate into changed behavior. Previous research supports the finding that understanding the consequences of sleep deprivation does not automatically lead to action [26,27,28,29,30,31]. Therefore, the knowledge gained in step 1 often remains abstract unless it is internalized as a personal concern. This highlights the need for a more culturally tailored approach in the subsequent steps to bridge the gap between awareness and action.

2. Step 2: Address the personal and cultural relevance of sleep. To move beyond mere knowledge, individuals must recognize sleep deprivation as a personal issue. This step builds on cultural values of self-care and well-being, which can help shift perceptions of sleep from a luxury to a necessary aspect of personal health. Integrating discussions of sleep as an integral part of one’s responsibilities—not only in terms of academic or work performance but also in preserving family harmony and societal productivity—can foster a greater commitment to improving sleep habits.

3. Step 3: Introduce three practical signs [88] to help individuals recognize sleep deprivation: (1) sleeping excessively on holidays, (2) feeling sleepy during the day on weekdays, and (3) falling asleep extremely quickly at night. Identifying these signs is important to help individuals recognize that they may be experiencing sleep deprivation. However, in Japan, acknowledging sleep deprivation can be seen as a weakness or failure to meet societal expectations. Therefore, creating an environment where recognizing and addressing sleep deprivation is culturally acceptable can help individuals transition from denial to action. For example, if an individual recognizes their sleep deprivation in step 3 and begins efforts to increase sleep time—such as recognizing that “getting 8 h of sleep will prevent me from falling asleep in class” or “if I sleep before midnight, I can wake up at 7:30 a.m.”—this can be considered an important achievement. This can be rephrased as recognizing the negative cycle of sleep deprivation (sleep deprivation → decreased concentration → reduced effectiveness → further work under wakefulness → sleep deprivation).

4. Step 4: Set sleep duration based on individual needs, taking into account cultural factors that influence daily routines and expectations. In Japan, rigid societal norms regarding work and study often lead to irregular sleep patterns. Therefore, instead of generalized age-based recommendations (Table 2) [89,90,91], presenting individualized OSD [92,93] can help guide individuals’ sleep decisions. This personalized approach (the details will be explained in Methodology section of this paper) respects cultural differences in sleep patterns and schedules while promoting healthier and more flexible sleep habits.

**Figure 1:**
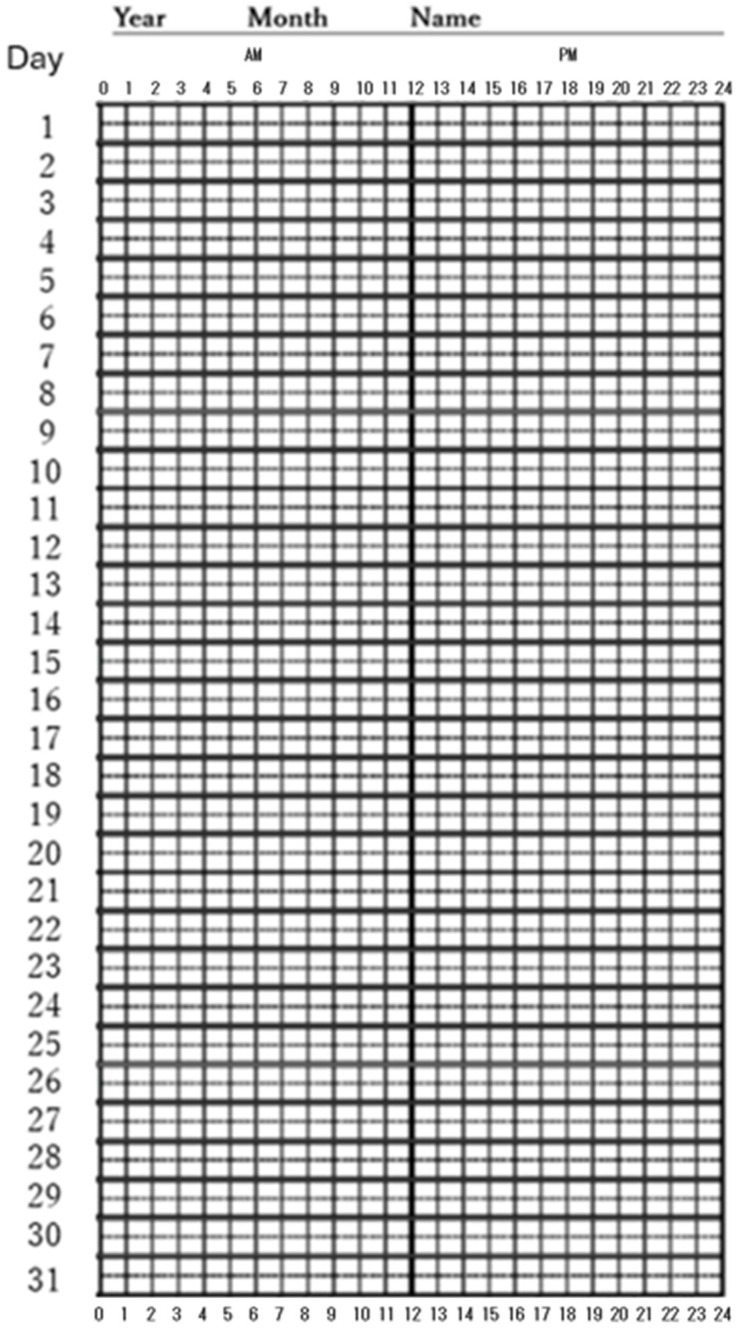
Sleep diary. Details on how to complete the log are provided in the text.

Another crucial method involves manually recording one’s sleep diary (Figure 1), rather than using computerized equipment [20]. This method helps individuals track and understand their sleep patterns, making the issue of sleep deprivation more tangible and personal. A sleep diary is to be recorded daily, with the horizontal axis showing midnight on the left and 24:00 on the right. In the upper section, a line is drawn to indicate sleep periods, and in the lower section, mealtimes and medication times [94] are recorded. This method enhances the cognitive–behavioral therapeutic value [20]. It is recommended to also record any instances of daytime dozing. Since the time frame is narrow, it is not necessary to record the exact time of sleep, whether it is 00:10 or 00:35, with meticulous attention; some flexibility is acceptable. However, if today is 20 April, and one is suddenly asked about bedtime on 11 April, it would likely be difficult to provide an accurate answer. While the exact time recorded each day can be approximate, it is essential that the log is filled out daily. Additionally, establishing a regular daily rhythm is encouraged, with breakfast intake recommended and late-night snacks discouraged [4,88]. As part of this process, individuals are also asked to record mealtimes in the sleep diary.

5. Step 5: Transform awareness into sustained change by emphasizing personal experience of achieving OSD. By experiencing this knowledge firsthand, individuals will deepen their understanding from step 2, and abstract knowledge will become more concrete through personal experience. In Japanese cultural narratives, sleeplessness is often associated with dedication and hard work, making it difficult for individuals to prioritize sleep without feeling guilty. By promoting personal experiences that demonstrate the value of well-rested states—such as improved mood, productivity, and overall well-being—individuals can internalize the importance of sleep. Once individuals experience firsthand the benefits of a full night’s sleep, they are more likely to reject the cultural narrative that sleep is secondary to work or academic achievement.

### Summary of Section 4

By incorporating these culturally relevant strategies, it is possible not only to raise awareness of sleep deprivation but also to instigate real behavioral changes that respect both personal well-being and cultural values. The integration of cultural context into these strategies is essential for achieving lasting changes in sleep health.

## 5. Methodology

To enhance step 4, the recently proposed simple formula for estimating individual OSD is introduced. Additionally, a more practical approach to evaluating OSD on a weekly basis, rather than daily, is presented.

The premise underlying the formula to assess individual daily OSD [92] is based on the observation of a U-shaped relationship between body mass index (BMI) and sleep duration [95], suggesting that individuals who achieve their OSD would not show significant deviations from the average BMI value of their population. Data from 2540 students in grades 5 through 11 (approximately ages 10 to 17) were analyzed [92,93]. Students who did not feel sleepy during class and whose BMI, standardized based on gender and grade, fell within ±1.5 were classified as “ideal students” (id-St). The “non-ideal students” (non-di-St) were categorized into 11 groups based on sleepiness scores (1 = never feel sleepy, 2 = occasionally, 3 = frequently, 4 = always) and standardized BMI (high (≥1.5), medium (±1.5), low (≤−1.5)).

The difference in average sleep duration between ideal students and each of these non-ideal student groups was added to the habitual sleep duration (HSD) of each non-ideal student, and their “assumed daily OSD” was calculated. A multiple regression model was then computed using the least squares method to predict the “estimated OSD”, with the “assumed daily OSD” as the dependent variable and the following explanatory variables: grade, gender, sleepiness score, actual BMI, self-reported academic performance, after-school activity (hours/week), breakfast frequency score, defecation frequency score, physical activity (days/week), screen time (on both schooldays and non-schooldays), bedtimes before both schooldays and non-schooldays, and waking times on schooldays and non-schooldays [91,92]. In this calculation, bedtimes and wake times were expressed in decimal hours. For example, bedtimes of 11:45 p.m. (23:45), 12:15 a.m. (0:15), and 3:15 a.m. (3:15) were represented as 23.75, 24.25, and 27.25, respectively, while wake times of 6:30 a.m. (6:30) and 11:00 a.m. (11:00) were represented as 6.50 and 11.00, respectively.

A total of 666 ideal students (id-St) were identified, and it was found that their average weekly sleep time was longer than that of non-id-St students: 62.0 h for elementary school students (compared to 61.2 h for non-id-St), 55.6 h for middle school students (compared to 54.1 h for non-id-St), and 50.1 h for high school students (compared to 48.6 h for non-id-St). All of these values exceeded the National Sleep Foundation’s lower range for an appropriate weekly sleep duration (49 h per week for children aged 6–17) [89]. Two key findings were confirmed: first, that the sleep duration of id-St students was within an appropriate range, and second, that id-St students slept longer than non-id-St students. The difference in average daily sleep duration between the id-St group (8.49 h) and the 11 non-id-St groups ranged from 0.04 h to 1.92 h.

A highly predictive linear equation was formulated to predict “estimated OSD” (adjusted R^2^ = 0.996, *p* < 0.001): 23.375 − 0.710 × (bedtime before schooldays) − 0.286 × (bedtime before non-schooldays) + 0.714 × (wake time on schooldays) + 0.281 × (wake time on non-schooldays) + 0.513 × (sleepiness score; 1–4) + 0.009 × [gender (male: 1; female: 2)] + 0.003 × (BMI). This equation facilitates the straightforward estimation of individual OSD levels based on wake-up times on schooldays and non-schooldays, bedtime before schooldays and non-schooldays, sleepiness during class, gender, and BMI [92]. Unlike the prior formula [93], which involved standardized BMI and social jetlag, increasing computational complexity, the updated formula simplifies these calculations. The equation indicates that individual daily OSD is positively correlated with earlier bedtimes, later wake times, increased sleepiness, female gender, and higher BMI. Regarding gender differences in OSD, this formula aligns with the findings of Franco et al. [96], suggesting that girls tend to sleep longer compared to boys from infancy to adolescence. Though the formula for estimating OSD provides promising insights into personalized sleep education, ongoing research is necessary to refine these methods and ensure their applicability across diverse demographic groups.

Another key aspect is the evaluation of OSD on a weekly, rather than daily, basis. In the previously discussed equation and in Table 2, OSD is presented on a daily basis, but modern lifestyles, particularly among adolescent populations, involve accumulating sleep debt during weekdays (the difference between HSD and OSD, i.e., the amount of sleep lost) and typically compensating for this sleep debt by sleeping in on weekends or holidays. This “weekend sleep-in” is not simply a way to “catch up on sleep”, but rather a means of repaying sleep debt accumulated during weekdays (sleep compensation). From a sleep science perspective, maintaining a consistent rhythm is ideal, but this is often unrealistic. Therefore, it is more practical to understand appropriate sleep time on a weekly rather than a daily basis. To demonstrate this concretely, for example, assuming the daily OSD derived from Table 2 or the proposed formula is 8 h, the weekly total would be 56 h. If an individual wants to sleep 1.5 h longer on weekends than on weekdays, the calculation would be 56 − (1.5 × 2) = 53 h. Dividing this by 7 gives an average of 7 h 34 min on weekdays and 9 h 4 min on weekends.

## 6. Future Prospects

The future directions for addressing sleep deprivation in children and adolescents will be discussed in two parts: a general perspective and a perspective specific to Japan.

Behavioral sleep interventions for children and adolescents have faced criticism for not being widely implemented [97]. These interventions include sleep hygiene education (guidance on good sleep habits, corresponding to the proposed step 1), relaxation techniques (such as progressive muscle relaxation and deep breathing), and cognitive behavioral therapy for insomnia, all aimed at addressing the thoughts and behaviors affecting sleep. These approaches are designed to improve sleep patterns and reduce stress. Zhou et al. [98] have pointed out that engaging in physical activity can help alleviate sleep problems in adolescents. Poor sleep habits are often linked to low socioeconomic status [99] and low life satisfaction [100]. Additionally, factors such as mood, stress, and exposure to light at night are key influencers of adolescent sleep, health, and well-being [101]. Reducing exposure to artificial light at night is particularly important for adolescents, as their lenses are more transparent and sensitive to light [102]. By implementing these strategies, adolescents may be able to improve their sleep and reduce stress [101]. The inclusion of the sleep diary, as introduced in the strategies section, could also be considered in future educational programs. However, its effective interpretation requires a certain level of competence, particularly in understanding the biopsychosocial model [4,103], which is closely related to sleep. Training consultants in this regard presents a significant challenge [47].

This review critically examines the negative impact of Bushidō on sleep practices in Japan. While Bushidō certainly embodies many virtues [64], it is also clear that, as discussed in this paper, it presents certain challenges. It should also be noted that the “dedicated teacher image” [104] refers to the concept of teachers who selflessly and passionately serve children. This ideal, which emerged in the 1920s, came to be associated with the necessity of self-sacrificial work. Postwar Japanese teachers consistently viewed themselves as “individuals who must be busy”, finding their identity in this busyness, which became central to the “dedicated teacher image”. The ideal of self-sacrifice, which this paper has discussed in relation to sleep deprivation, has deeply permeated the mindset of Japanese teachers. To eliminate the harmful sleep-related practices that have become widespread in Japan, it is essential not only for school and educational professionals but also for all individuals involved in the future of the next generation to accurately understand the challenges posed by the excessive self-sacrifice promoted by Bushidō. Furthermore, it is necessary to recognize the cultural issues associated with the tendency to prioritize activities over sleep, and to effectively eliminate these practices. While this may take time, I believe it is crucial to widely disseminate the commonsense notion that “everyone should know the amount of sleep they need (=OSD), adjust their sleep on a weekly basis, and if still feeling sleepy, simply sleep”. I also feel that persistent efforts from many people will be required to achieve this goal.

Finally, children and adolescents with neurodevelopmental or medical issues, who are at higher risk of sleep disturbances, have not been sufficiently addressed in sleep interventions [81,105,106,107]. Approaches to sleep disorders in these groups remain unresolved.

## 7. Conclusions

1. The undervaluation of sleep in Japan is hypothesized to stem from the cultural aesthetics of self-sacrifice and asceticism, particularly as embodied in the Bushidō spirit.
2. Overcoming this deeply rooted aesthetic requires a critical evaluation of Bushidō’s limitations in modern society, rather than its uncritical veneration. These insights must be critically examined and effectively transmitted to future generations.
3. A pivotal factor in promoting adequate sleep is the individual’s direct experience of recovery from sleep deprivation—specifically, recognizing the resolution of associated difficulties and the benefits of improved sleep.
4. For children and adolescents, a key educational task is the introduction of methods to calculate each individual’s OSD, which may help foster awareness of personal sleep needs.
5. Further research and replication studies are needed; however, the proposed formula for estimating individual OSD uses only bedtime on school nights and non-school nights, wake-up times on schooldays and non-schooldays, sleepiness, gender, and BMI. This formula is non-invasive, requires no special equipment, and holds promise for practical application.
6. Since adolescents accumulate sleep debt during weekdays and compensate for this debt on weekends or holidays, it is more practical to consider appropriate sleep time on a weekly rather than a daily basis.
7. This review underscores the critical need for cultural transformation in Japan’s approach to sleep health, while also advocating for individualized sleep strategies to improve sleep health literacy.
8. While this review highlights Bushidō-related values—such as self-sacrifice and asceticism—as cultural factors contributing to sleep deprivation in Japan, it is equally important to recognize the impact of contemporary influences, including electronic device use, academic pressure, and sedentary behavior. Addressing sleep deprivation among youth requires a comprehensive, multifactorial approach, and the present discussion offers one interpretive framework among many.

## Figures and Tables

**Table 1:** Five critical steps necessary to fostering awareness that transcends self-sacrifice and asceticism for reducing sleep deprivation.

Step	Goal	Explanation	Difficulty
1	Acquiring knowledge about the detrimental effects of sleep deprivation	This is similar to the basics of widely implemented programs.	Easy
2	Achieving personal relevance regarding the harms of sleep	It is difficult to reach this awareness with step 1 alone. In such cases, consider progressing to step 3.	Hard
3	Understanding three simple indicators of sleep deprivation	As these are concrete indicators, they can serve as significant triggers for self-awareness of sleep deprivation.	Slightly hard
4	Monitoring one’s own sleep duration	This is a step further after step 3. Once this step is reached, problem-solving is within sight.	Relatively easy, if step 3 is cleared
5	Confirming step 2	After recognizing one’s optimal sleep duration on a weekly basis, the abnormality of previous sleep patterns can be recognized more strongly and impressively. This step further reinforces self-awareness regarding the issues of sleep deprivation.	Easy

**Table 2:** Sleep duration recommendations (in hours) for children and adolescents.

National Sleep Foundation [89]				American Academy of Sleep Medicine [90]		Centers for Disease Control and Prevention [91]	
Age	MBA	Rec	MBA	Age	Rec	Age	Rec
6–13	7–8≤	9–11	≤12	6–12	9–12	6–12	9–12
14–17	7≤	8–10	≤11	13–18	8–10	13–17	8–10
18–25	6≤	7–9	≤10–11			18–60	7 or more

MBA: may be appropriate; Rec: recommendation.

## Data Availability

No new data were created or analysed in this study. Data sharing is not applicable to this article.
